# Tackling anti-tumor macrophage function through genome engineering

**DOI:** 10.1016/j.omton.2026.201304

**Published:** 2026-08-13

**Authors:** Richa Kaushal, Joseph G. Skeate, Andrew T. Crane

**Affiliations:** 1 Department of Medicine, Division of Gastroenterology, Hepatology, and Nutrition, University of Minnesota, Minneapolis, MN, USA; 2 Department of Pediatrics, Division of Hematology/Oncology, University of Minnesota, Minneapolis, MN, USA; 3 Masonic Cancer Center, University of Minnesota, Minneapolis, MN, USA; 4 Center for Genome Engineering, University of Minnesota, Minneapolis, MN, USA; 5 Stem Cell Institute, University of Minnesota, Minneapolis, MN, USA

## Main text

**Figure 1:**
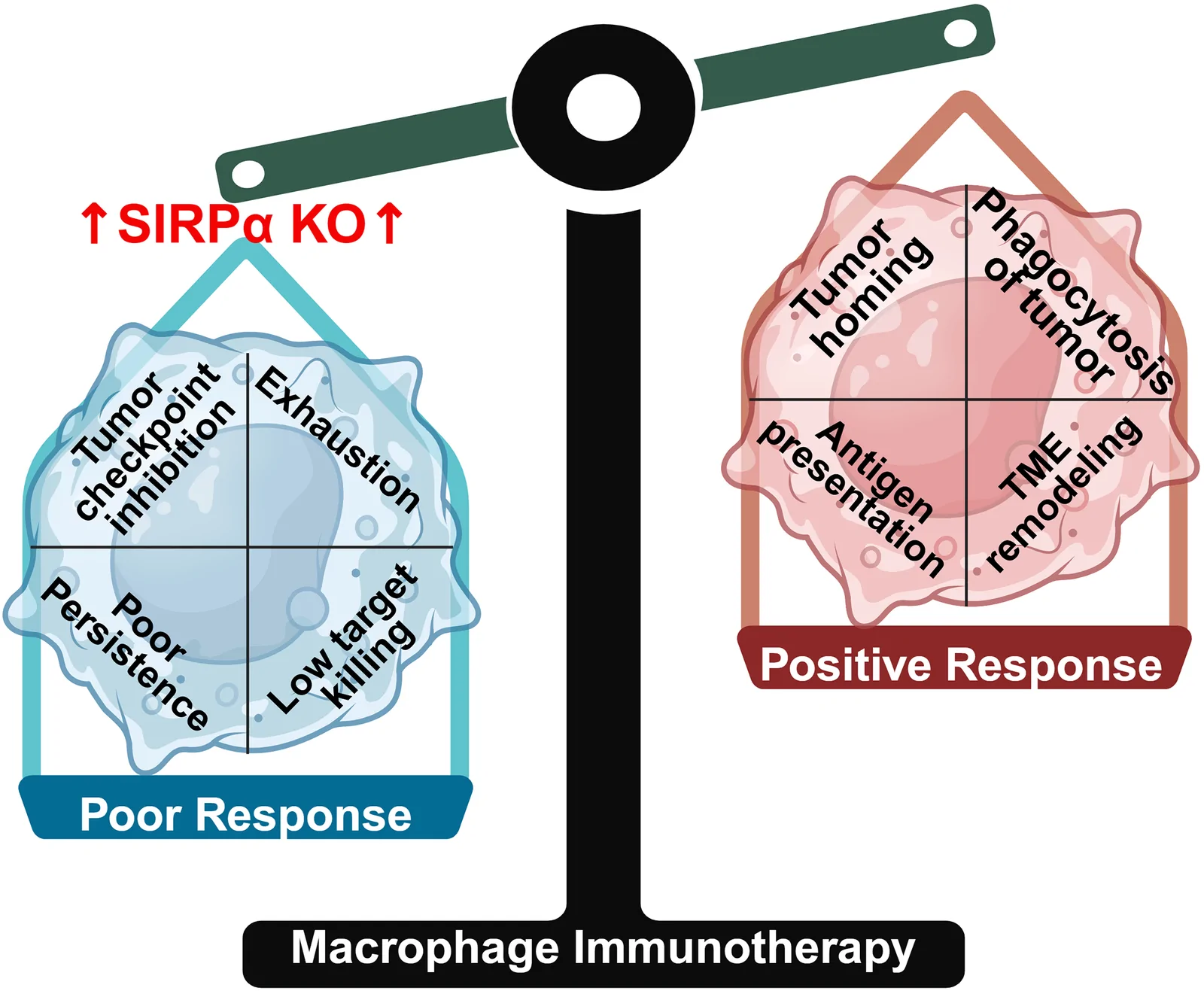
The benefits of macrophage immunotherapy are still outweighed by the tumor microenvironment suppressing effector function Genome editing to disrupt SIRPα expression changes the balance slightly in favor of anti-tumor macrophages. Image was created using Biorender.com.

In the realm of cellular immunotherapies, the synthetic chimeric antigen receptor (CAR) has proven to be a revolutionary modality, leading to the US Food and Drug Administration approval of multiple autologous CAR T cell products. Despite their remarkable success in hematological malignancies, CAR T cell therapies have shown limited efficacy in solid tumors, prompting worldwide efforts to extend CAR engineering to alternative immune effector cells, including macrophages. Macrophages possess inherent qualities that are beneficial for immunotherapy, including tumor homing, clearance of malignant cells through phagocytosis, and initiation of an adaptive immune response through cytokine/chemokine signaling and antigen presentation. These benefits, however, are outweighed by the tumor microenvironment (TME) and tumor-macrophage checkpoints that limit overall response to macrophage immunotherapies (Figure 1). In an effort to change the balance for heightened anti-tumor effects, a recent publication in *Molecular Therapy Oncology* by Smith and colleagues disrupted the “don’t eat me” CD47-SIRPα axis using CRISPR-Cas9-mediated knockout (KO) of SIRPα on induced pluripotent stem cell (iPSC)-derived macrophages (iMacs).^1^ SIRPα KO iMacs demonstrated significantly higher engagement and phagocytosis of tumor cell lines in repeated stimulation assays, indicating resistance to phagocytosis-induced exhaustion. This strategy proved effective when tumor cells were opsonized with clinically relevant monoclonal antibodies as well as when combined with CAR engineering. This study adds to the mounting body of literature suggesting that KO of SIRPα is necessary for future macrophage-based cancer immunotherapies, not only to improve tumor engagement but also to preserve macrophage fitness.

The CD47-SIRPα axis has long been understood as one of many pathways by which tumors evade immune surveillance by macrophages. The SIRPα receptor on macrophages interacts with the transmembrane CD47 protein, resulting in an inhibition of activation and phagocytosis.^2^ On most malignant cells, CD47 is highly expressed, and combined with other TME cues, macrophages adopt a tumor-associated macrophage phenotype to promote tumor growth and metastasis. Disrupting this axis through pharmacological or biologic mediators has demonstrated robust pre-clinical efficacy, yet clinical translation did not meet expectations.^3^ Unlike systemic CD47 blockade, permanent disruption of SIRPα in a therapeutic cell product avoids continuous antibody administration while potentially reducing harmful on-target, off-tumor interactions. Other approaches to blocking the CD47-SIRPα axis in cellular therapies have included a synthetic tumor targeting transgene with built-in secreted CD47 blockers^4^ or *in vivo* RNA silencing of SIRPα.^5^ Using genome editors, we have previously demonstrated efficient disruption of SIRPα expression in primary human monocytes using CRISPR-Cas9, enhancing phagocytosis of tumor cells *in vitro*.^6^ Smith and colleagues expanded upon this finding to investigate both *in vitro* and *in vivo* efficacy. When combined with anti-HER2 or anti-GD2 monoclonal antibodies, SIRPα KO iMacs phagocytosed at significantly higher levels than non-engineered parental iMacs and maintained functionality in a serial killing assay. Using an orthotopic ovarian cancer model, the combination of an anti-HER2 monoclonal antibody with SIRPα-KO iMacs prolonged overall survival. These results highlight previously uninvestigated mechanisms of SIRPα, including downregulation of genes related to anti-inflammatory tumor-associated macrophages.

Although the disruption of the CD47-SIRPα axis enhanced macrophage function, significant barriers remain for macrophage-based cellular immunotherapies, including *in vivo* persistence, dosing, and a highly immunosuppressive TME. Results of a clinical trial sponsored by the now shuttered Carisma Therapeutics highlight these limitations, with only a subset of patients demonstrating a mean progression-free survival of 8 weeks.^7^ This correlated with a rapid decline in CAR-expressing macrophages in the TME 4 weeks after infusion. It is possible that a lack of persistence can be overcome by repeated dosing of engineered CAR macrophages, but it does not yet address that a high effector-to-target ratio (5 SIRPα KO iMacs to 1 tumor cell used by Smith and colleagues)^1^ is seemingly required to demonstrate a significant anti-tumor response. Repeated dosing adds manufacturing, logistical, and financial burdens to patients, families, and caregivers, while potentially increasing the risk of treatment-associated adverse events.

Historically, primary human myeloid lineage cells have been resistant to genome engineering due to innate DNA and RNA sensing mechanisms and limited *ex vivo* expansion. By reverting to the near-limitless resource of iPSCs, it is possible to perform complex genomic alterations with single-clone expansion and targeted differentiation to derive sufficient quantities of a cellular product for immunotherapy. For SIRPα disruption, Smith and colleagues designed single-guide RNAs to span the CD47-binding domain of exon 3. An individual clone from the bulk CRISPR-Cas9-edited population was selected, with genomic confirmation of roughly 300-bp deletion of the target region. Clonal selection, at this stage, allows for a more in-depth analysis of potential chromosomal alterations, which is not feasible in bulk populations isolated from primary human cells.

Macrophages are key mediators of the adaptive immune response, with machinery for antigen presentation and cytokine/chemokine expression for T and NK cell activation that is downregulated within the TME.^8^ Elevated expression of the inflammatory genes IL-1B and IL-6 was observed following co-culture of SIRPα-KO iMacs with tumor targets, but it remained unclear if this transcriptional activation translates to sufficient cytokine production to induce a robust host-immune response against the tumor. Clinical data suggest that CAR-macrophage mediated expression of pro-inflammatory cytokines was insufficient to induce a significant T cell response, which could be attributed to other factors of tumor-mediated immune suppression.^7^

Identifying and addressing each of these key burdens will be necessary for a macrophage-based cellular immunotherapy to be clinically viable. Published reports on macrophage immunotherapies have demonstrated that the expression of a CAR can induce phagocytosis-mediated tumor clearance and neoantigen presentation and that the disruption of the CD47-SIRPα axis can improve macrophage fitness. Co-opting strategies from next-generation CAR T or CAR NK, such as cytokine armoring, engineered neoantigen presentation, or enhanced expression of inflammatory cytokines, may improve host lymphocyte infiltration and activation.^9^^,^^10^ Likely, engineering macrophages for cellular immunotherapy will require a combination of these tools to generate a robust anti-tumor response by the host. Furthermore, extensive genome modification will be required to immunologically cloak iMacs as they move closer to clinical trials to prevent allogeneic graft rejections. While the field of macrophage-based anti-cancer immunotherapy is still emerging, the findings by Smith and colleagues in disrupting the CD47-SIRPα axis is another step toward developing clinically viable cell products.

## Declaration of interests

The authors declare no competing interests.
